# Thermal Strain Detection for Concrete Structure Cold Shrinkage under Stress Constraint with FBG

**DOI:** 10.3390/s22249660

**Published:** 2022-12-09

**Authors:** Lubing Yang, Chuan Li, Chuan Luo

**Affiliations:** 1 Faculty of Information Engineering and Automation, Kunming University of Science and Technology, Kunming 650500, China; 2 Yunnan Key Laboratory of Computer Technology Applications, Kunming University of Science and Technology, Kunming 650500, China

**Keywords:** concrete structure, temperature load, stress load, FBG, thermal strain, cold shrinkage

## Abstract

Additional strain increments occur in concrete subject to stress constraints during cold shrinkage, resulting in irregular deformation and reducing the concrete structure’s stability. When an annular concrete structure is subjected to radial pressure, two tensile stress concentration zones will appear at the intersection of the inner wall and the diameter along the pressure direction. When exposed to low temperatures, the total strain in the tensile stress concentration zones is caused by the combined effect of applied stress strain and thermal strain. Then, the thermal strain of the structure can be obtained from the difference between the total strain and the applied stress strain. Gradient cooling was performed after applying radial pressure to the annular concrete using a counterforce device. The applied stress strain and total strain of the tensile stress concentration zones are measured by fiber Bragg grating (FBG) strain sensors fixed along the stress direction. According to the measurement results, the thermal strains of the concrete structure under the stress constraint are extracted to analyze the influence of the tensile stress constraint on the thermal strain of the concrete structure. In the temperature range of $-40{\;}^{\circ }C∼20{\;}^{\circ }C$, the thermal strains of the structure under radial pressures of 1500 N, 2000 N, and 3000 N are extracted, respectively. The thermal expansion coefficients are calculated based on the thermal strain of the structure. The free thermal expansion coefficient of concrete structures fluctuates around $11\times 10^{-6}{/}^{\circ }C$. When the temperature is reduced to $10{\;}^{\circ }C$, the difference between the thermal expansion coefficient under the stress constraint and the free thermal expansion coefficient is the largest. When the temperature is reduced to $-20{\;}^{\circ }C$, the thermal expansion coefficients under each stress condition are close to the same. The results show that the stress confinement significantly inhibits the cold shrinkage of the concrete structure, and the inhibitory effect is gradually weakened when the temperature decreases.

## 1. Introduction

When a concrete structure subject to tensile stresses is exposed to low temperatures, the cold shrinkage of the structure is constrained by the tensile stresses. On the one hand, additional strain increments are generated within the concrete structure due to cold shrinkage being restrained. On the other hand, since the axial tensile strength of the concrete structure is much less than the axial compressive strength [1,2,3], the tensile stress constraint will cause the concrete structure to be more prone to cracking during cold shrinkage.

Due to the uneven compression of the surrounding rock, significant tensile stress will occur at local locations in the lining structure of the tunnel [4,5,6,7]. Thus, it is common for tensile stresses to constrain the cold shrinkage of concrete structures in the lining structure of tunnels located in extremely cold regions. Until now, many reports have shown that damage to tunnel lining structures occurs in winter more frequently than in other seasons. Low temperature has become an extreme environmental factor of great concern, affecting the stability of concrete structures [8,9,10]. Yu Yuan et al. (2021) [11] deduced an analytical solution for the frost heave pressure of the surrounding rock and analyzed the circumferential stress distribution of the lining structure. The research results show that the lining structure was subject to uneven compression caused by the freezing depth difference of the surrounding rock, and bending tension occurred at the location of the tunnel vault. Hong Yu et al. (2021) [12] studied the stress variation of the tunnel secondary lining structure in different seasons by means of field monitoring and numerical analysis. The results show that the tensile stresses generated in the secondary lining were significantly greater in winter than in the warm season. The suppressed cold shrinkage of the lining structure can lead to larger thermal stresses within the concrete structure. Chuan Li et al. (2022) [13] analyzed the strain changes in Baima Snow Mountain’s lining structure over a period of one year based on the field monitoring results. The results show that tensile strain and thermal strains were significantly present within the lining structure in winter. The low temperature caused a significant degree of cold shrinkage in the lining structure. Xianzhang Ling et al. (2022) [14] analyzed the stress response of the tunnel lining structure subjected to frost heave of the surrounding rock by establishing a numerical model. The results show that the sides and bottom of the tunnel were subjected to tensile damage when the circular tunnel was squeezed by the frost heave of the surrounding rock. Testing and studying the cold shrinkage behavior of concrete structures under stress constraints can provide a theoretical basis for structural health analysis of tunnels in cold regions.

In the field of structural detection, FBG sensors are widely used for structural strain detection because of their wide measurement range, low transmission loss, high accuracy, and stable operation in harsh conditions [15,16,17,18,19]. Zhao Xuefeng et al. (2015) [20] used FBG sensors for residual strain detection during the freeze-thaw cycles of concrete structures. The feasibility of the FBG strain sensor for deformation detection during the freeze–thaw cycle of concrete structures was verified by comparing it to traditional measurement methods. Zhengwu Jiang et al. (2019) [21] embedded the FBG into cement materials to detect the thermal strain of the structure under low-temperature conditions. The feasibility of FBG in characterizing the thermal strain of cement materials under low-temperature conditions was verified by analyzing the relationship between the freezing process of pore water and thermal strain.

The tunnel lining is a typical annular concrete structure. Thus, the annular concrete structure was used to study the effect of tensile stress constraint on structural cold shrinkage in this paper. By applying radial pressure to the annular concrete structure, tensile stresses were created at local locations in the structure. Gradient cooling was applied to the annular concrete structure after applying radial pressure. The applied stress strain and total strain of the structure were measured using FBG strain sensors to extract the thermal strain of the concrete structure under a tensile stress constraint. The thermal expansion coefficients of the concrete structure under different stress conditions were calculated to analyze the effect of tensile stress constraint on the cold shrinkage of the structure.

## 2. Principle of Thermal Strain Detection for Concrete Structures under Stress Confinement with FBG

**Figure 1:**
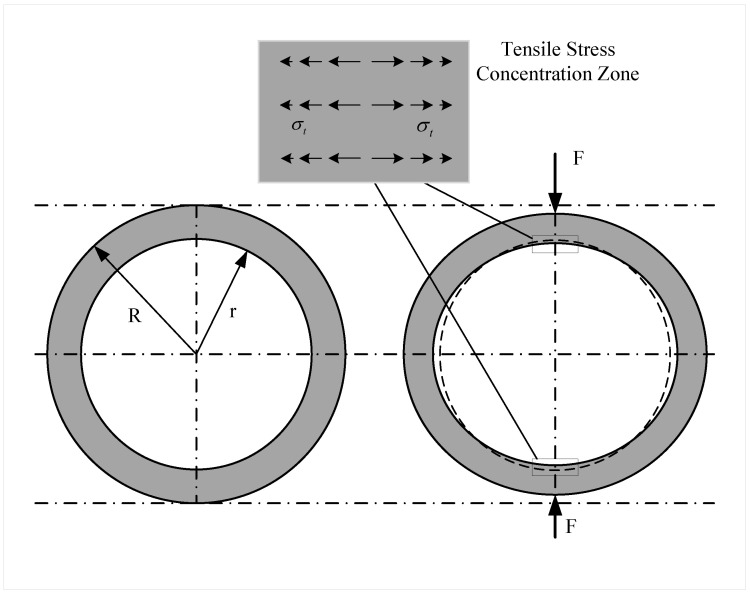
Diagram of ring structure deformation under radial pressure.

When the annular concrete structure is subjected to radial pressure, the structure shrinks along the direction of the pressure and stretches along the direction perpendicular to the pressure, as shown in Figure 1. Deformation of the structure will lead to significant bending tension at the intersection of the inner wall and the diameter along the pressure direction. Bending tension can lead to tensile stresses within the structure. Thus, two tensile stress concentration zones will appear in the inner wall of the structure [22,23,24].

When the structure is subjected to radial pressure *F*, the tensile stress $\sigma_{t}$ at the intersection of the diameter along the direction of pressure and the inner wall can be expressed as [25]:

(1) $$\sigma_{t}=\frac{F}{\pi Rt}\left(6+38{\left(\frac{r}{R}\right)}^{2}\right)$$

where *r* is the internal radius, *R* is the external radius of the structural, and *t* is the thickness of the structural. When the structure is exposed to an ultra-low temperature environment, the stress concentration zones are subjected to the combined effect of external load and temperature load, and the total strain can be expressed as:

(2) $$\varepsilon_{\mathrm{tot}}=\varepsilon_{T}+\varepsilon_{W}$$

where $\varepsilon_{\mathrm{tot}}$ is the total strain of the structure during the cooling process, $\varepsilon_{T}$ is the thermal strain of the structure during the cooling process and $\varepsilon_{W}$ is the applied stress strain. The applied stress strain $\varepsilon_{W}$ can be expressed as:

(3) $$\varepsilon_{W}=\frac{\sigma_{t}}{E}$$

Thus, the thermal strain of the structure under stress constraint can be obtained from the difference between the total strain and the applied stress strain as follows:

(4) $$\varepsilon_{T}=\varepsilon_{\mathrm{tot}}-\frac{\sigma_{t}}{E}$$

where *E* is the Young’s modulus of the structure.

**Figure 2:**
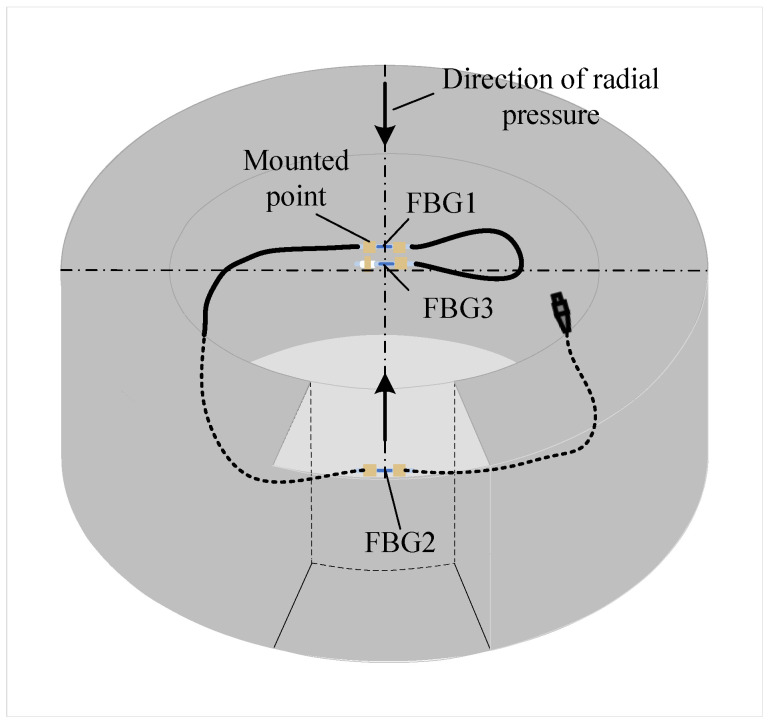
Diagram of strain detection of the structure with FBG.

The FBG strain sensors are fixed in the stress concentration region along the stress direction for measuring total and applied stress strains, as shown in Figure 2. FBG1 and FBG2 are fixed at both ends of the structure surface, and these two sensors are used to detect structural strains. FBG3 is fixed at one end to the structure surface and suspended at the other end for temperature compensation of FBG1 and FBG2. When the structure subjected to radial pressure is exposed to low temperatures, the center wavelengths of FBG1 and FBG2 are modulated by both the ambient temperature and the structure strain. The center wavelength of FBG3 is modulated by the ambient temperature only. Therefore, the wavelength signals of FBG1 and FBG2 carry the structural strain and ambient temperature information. The wavelength signal of FBG3 carries the ambient temperature information.

According to the principle that the center wavelengths of FBG1, FBG2, and FBG3 are modulated by different external parameters, the Bragg grating center wavelength shift $\Delta \lambda_{g}$ and $\Delta \lambda_{T}$ of the two FBGs for strain detection and the FBG for temperature compensation can be described as:

(5) $$\left\{\begin{array}{c} \Delta \lambda_{g}=S_{\varepsilon }C_{g,\varepsilon }\varepsilon +S_{T}\Delta T\\ \Delta \lambda_{T}=S_{T}\Delta T \end{array}\right.$$

where ε is the strain variation of the structure, ΔT is the temperature variation, and $C_{g,\varepsilon }\approx 0.98$ is the strain transmission coefficient between the FBG and the structure. At 1550 nm, the strain and thermal sensitivities are $S_{\varepsilon }\approx 1.21\;\mathrm{pm}/\mu \varepsilon$ and $S_{T}\approx 10.3\;\mathrm{pm}/{}^{\circ }$C for silica-based fiber Bragg grating. According to Equation $\left(5\right)$, the temperature variation ΔT and the strain of the structure ε can be described by

(6) $$\left\{\begin{array}{c} \varepsilon =\frac{\Delta \lambda_{g}-\Delta \lambda_{T}}{S_{\varepsilon }C_{g,\varepsilon }}\\ \Delta T=\frac{\Delta \lambda_{T}}{S_{T}} \end{array}\right.$$

The concrete structural strain measured by the FBG strain sensor includes two parts: applied stress strain and thermal strain. Combining the Equations $\left(4\right)$ and $\left(6\right)$, the thermal strain $\varepsilon_{T}$ of the structure can be described by:

(7) $$\varepsilon_{T}=\frac{\Delta \lambda_{g}-\Delta \lambda_{T}}{S_{\varepsilon }C_{g,\varepsilon }}-\varepsilon_{W}$$

## 3. Experimental Setup and Methods

**Figure 3:**
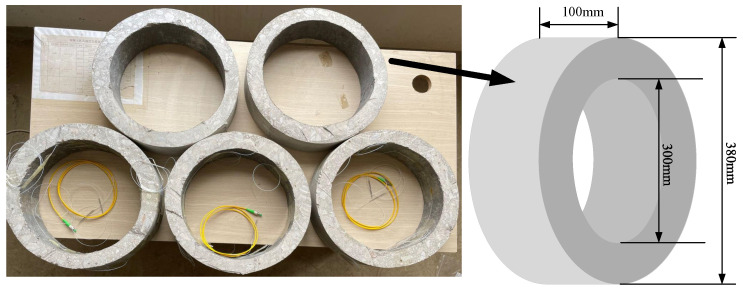
Diagram of concrete specimen.

The annular concrete structures with an inner diameter of 300 mm and an outer diameter of 380 mm are fabricated as shown in Figure 3. The annular concrete structures are made by concrete centrifuge, and the structures are placed in a curing room for standard maintenance for 28 days after the molds were removed. The temperature is $20{\;}^{\circ }C$ and the relative humidity is 97%. Three cubic concrete blocks $\left(150\;\mathrm{mm}\times 150\;\mathrm{mm}\times 150\;\mathrm{mm}\right)$ are made according to the mix ratio of concrete pipe batching for mechanical properties testing. The relevant mechanical properties of concrete structures are compressive strength, $\sigma_{c}\;=\;19.1\;N/mm^{2}$; Young’s modulus, $E=1.2\times 10^{4}\;N/mm^{2}$; tensile strength, $\sigma_{t}\;=\;1.83\;N/mm^{2}$; Poisson’s ratio, v=0.2.

**Figure 4:**
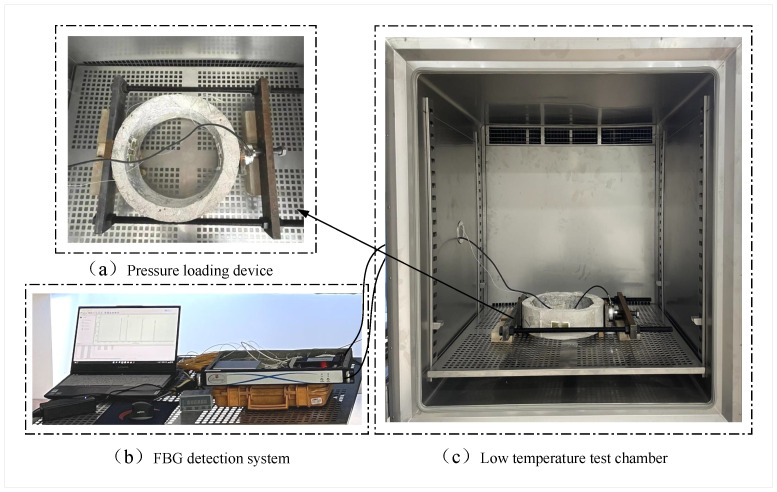
Diagram of experimental setup.

The experimental setup is composed of an FBG detection system, low-temperature test chamber, and pressure loading device, as shown in Figure 4. First, the pressure loading device is used to apply radial pressure to the concrete structure, and the FBG detection system is used to detect the applied stress strain in the two stress concentration zones under radial pressure. Then, the structure is cooled down in a gradient using the low-temperature test chamber and the total strain of the structure during the cooling process is measured. The operating temperature range of the low-temperature chamber is $160∼-60\;{}^{\circ }$C, with an error of $\pm 2\;{}^{\circ }$C, as shown in Figure 4a.

**Figure 5:**
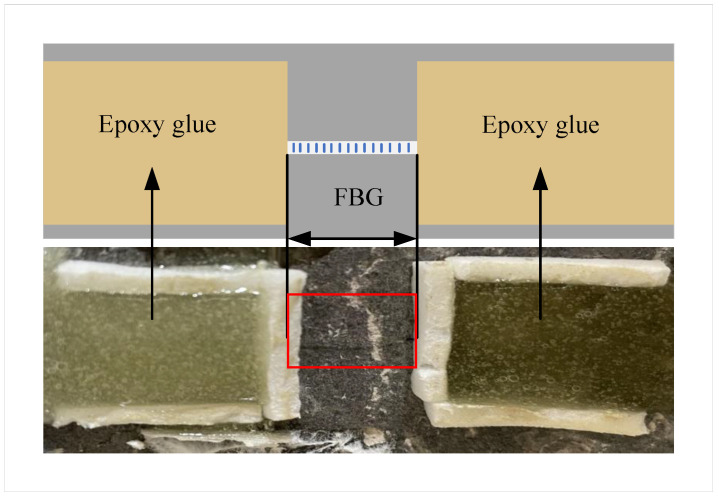
Diagram of the FBG strain sensor fixed on the structure.

The FBG detection system is composed of an FBG sensor, FBG demodulation device, and main unit, as shown in Figure 4b. The FBG is fixed to the structure and reflects the wavelength signal of the light modulated by the structural strain and temperature. The FBG’s grating length is 10 mm, the bandwidth is ≤0.3 nm, the reflectivity is ≥90%, and the SLSR is ≥15 dB. The reflected light signal enters the demodulation device through the circulator. The demodulation device detects and outputs the center wavelength of the reflected light signal. The ends of the FBG are glued to the stress concentration area along the stress direction, as shown in Figure 5. Due to the small area of stress concentration generated by the structure under diameter pressure, it is necessary to ensure that the effective measurement length of the grating is as short as possible. Therefore, the mounting points at the ends of the FBG need to be as close to the FBG section as possible. This way of installing FBG can effectively reduce the strain transfer loss caused by partial deformation of the fiber, and improve the strain transfer efficiency between the grating and the structure.

**Figure 6:**
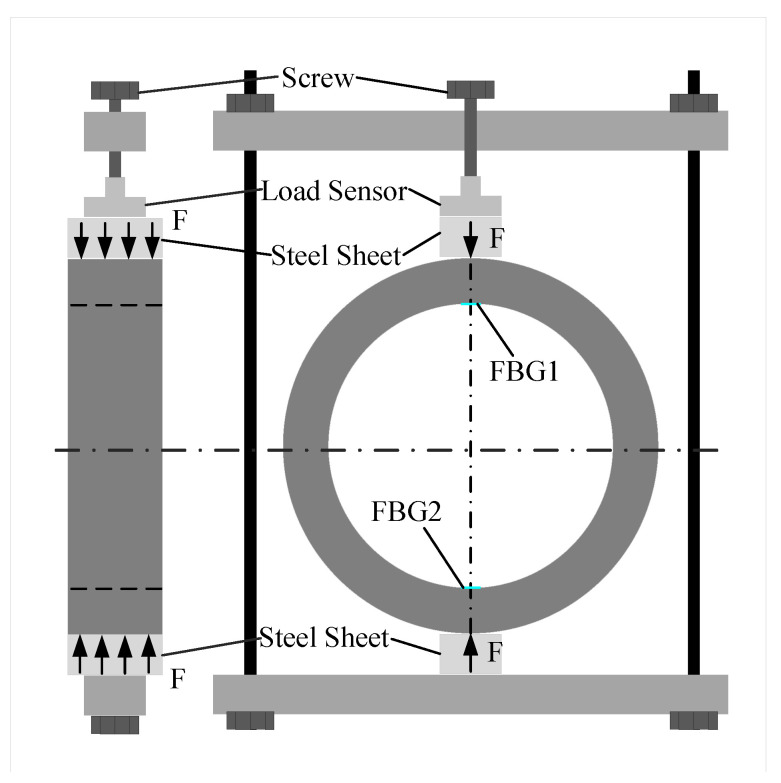
Diagram of pressure loading.

The radial pressure loading device consists of a counterforce frame and a load sensor, as shown in Figure 4a. The concrete specimen is placed flat during the experiments, which can effectively overcome the effect of self-weight on the deformation of the structure, and ensure that the stress condition in the two stress concentration zones is consistent. By rotating the screw to apply pressure to the load sensor, the pressure is transferred from the load cell to the steel plate, which squeezes the structure and finally forms a line load at the contact position between the steel plate and the structure, as shown in Figure 6. In this case, the load sensor is not only used to transmit the pressure, but also to display the pressure value. During the cooling process, the pressure on the structure increases due to the thermal expansion coefficient mismatch between the reaction frame device and the concrete structure. Therefore, the pressure needs to be adjusted according to the load sensor indication during the experiment. The related technical parameters for the load sensor can be seen in Table 1.

A strain calibration experiment on the FBG strain sensor is performed by applying different loads at both ends of the sensor. The strain sensitivity coefficient of the FBG strain sensor is 1.2 pm/με and the temperature sensitivity coefficient of the FBG used for temperature compensation is 8.4 pm/${}^{\circ }C$.

Demarcated by experiments, the system of Equation $\left(6\right)$ can be expressed as

(8) $$\left\{\begin{array}{c} \varepsilon =\frac{\Delta \lambda_{g}-\Delta \lambda_{T}}{1.2}\\ \Delta T=\frac{\Delta \lambda_{T}}{8.4} \end{array}\right.$$

## 4. Analysis of Experimental Results

### 4.1. Applied Stress-Strain Analysis of the Structure under Radial Pressure

The applied stress strain in the stress concentration zone of the annular concrete structure under radial pressure is tested experimentally. In order to detect the strain change in the structure during cold shrinkage, the structure needs to be kept undamaged during the experiment. Therefore, the pressure applied to the specimen needs to be less than its radial pressure at the ultimate fracture. The ultimate radial pressure *F* of the specimen structure is obtained from Equation $\left(1\right):$

(9) $$F=\frac{\sigma_{x}}{\pi Rt}\left[6+38{\left(\frac{r}{R}\right)}^{2}\right]$$

where $\sigma_{x}$$\left(\mathrm{MPa}\right)$ is the tensile strength of the concrete structure. Substituting the relevant parameters of the specimen into Equation (1), the ultimate radial pressure of the structure was obtained as 3509 N. Therefore, the radial pressures applied to the structure were selected as 1500 N, 2000 N, and 3000 N in the experiment. Combining Equations $\left(1\right)$ and $\left(3\right)$, the applied stress strain $\varepsilon_{W}$ in the two stress concentration zones of the structure under radial pressure can be described by:

(10) $$\varepsilon_{W}=\frac{\sigma_{t}}{E}=\frac{F}{\pi RtE}\left(6+38{\left(\frac{r}{R}\right)}^{2}\right)$$

**Figure 7:**
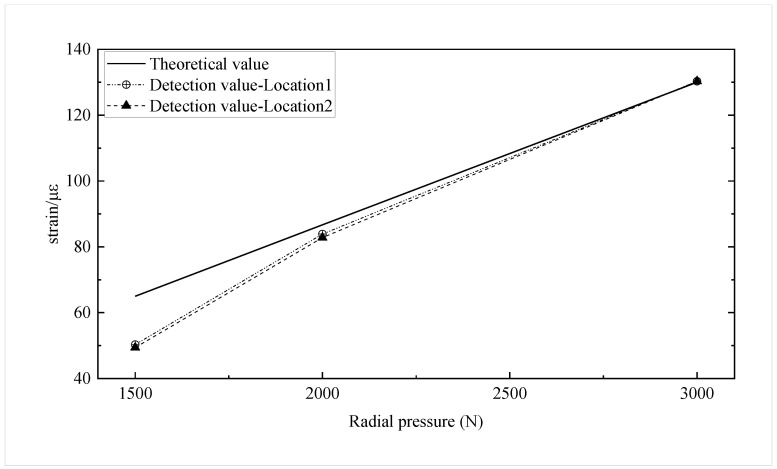
Theoretical and detection values of applied stress strain at each radial pressure.

The stresses in the tensile stress concentration zone under the above pressure conditions are calculated to be $0.78\;N/mm^{2}$, $1.04\;N/mm^{2}$, and $1.56\;N/mm^{2}$, respectively. The applied stress strains of the two testing locations are measured using FBG strain sensors under radial pressures of 1500 N, 2000 N, and 3000 N, respectively. The theoretical and detection values of the applied stress strain for each stress condition are shown in Figure 7.

The detected values of applied stress strain at 2000 N and 3000 N radial pressure are close to the theoretical values. The detected values of applied stress strain 1500 N radial pressure are less than the theoretical value. The error may be caused by the fact that the radial pressure is slight, and the deformation of the concrete structure is in the compaction stage before it enters the elastic zone [26]. The measured applied stress-strain trends in the two stress concentration zones are consistent with the theoretical calculations.

### 4.2. State Analysis of FBG in Structural Strain Detection

**Figure 8:**
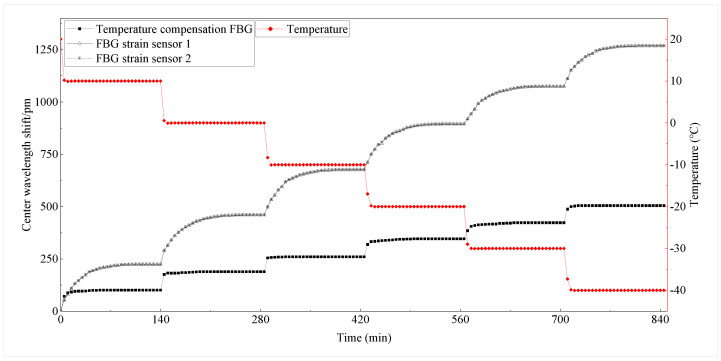
Center wavelength shift of FBG and temperature in the experimental chamber.

Gradient cooling of the structure under stress constraint is performed in the temperature range of $-40{\;}^{\circ }C∼20{\;}^{\circ }C$, the initial temperature is $20{\;}^{\circ }C$, and the temperature increment is $-10{\;}^{\circ }C$. The temperature inside the test chamber, the center wavelength shift of the temperature-compensated FBG, and the center wavelength shift of the FBG strain sensors are recorded in the experiment, as shown in Figure 8. The center wavelength shift of the FBG strain sensors and the temperature-compensated FBG gradually increase as the temperature decreases. Moreover, the difference between the center wavelength offset of the two FBGs gradually increases as the temperature decreases.

The center wavelength of the temperature-compensated FBG is only modulated by temperature. Thus, the center wavelength variation will tend to be stable with the temperature change in the cryostat within a short period. The center wavelength of the FBG strain sensor is modulated by both temperature and structural strain, and the thermal strain of the concrete structure is a slowly changing process. Thus, the center wavelength of the FBG strain sensor needs to stabilize over a certain period. Precisely, the concrete structure is cooled by convective heat transfer and heat conduction with the air inside the test chamber, which results in the time required for the overall temperature of the structure to reach the preset temperature. The above results indicate that the structural strain modulates the central wavelength shift of the FBG strain sensor. The FBG strain sensor captures the strain in the concrete structure.

### 4.3. Extraction of Structural Thermal Strain

**Figure 9:**
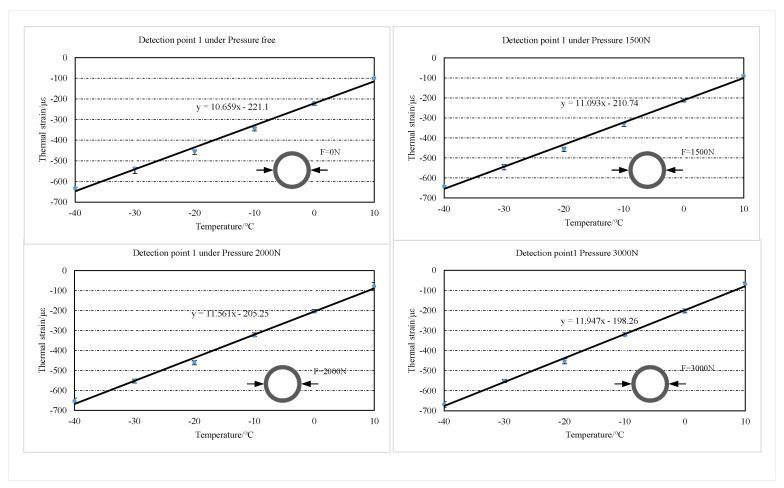
Error bar of the thermal strain in detection point 1 under each stress condition.

**Figure 10:**
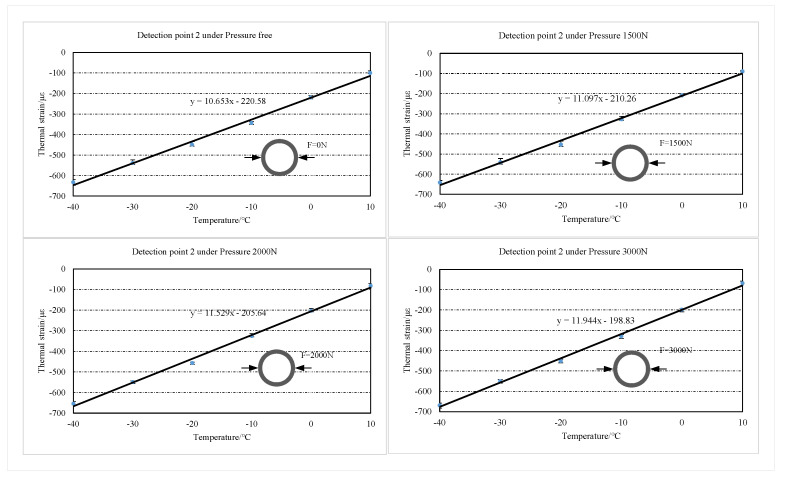
Error bar of the thermal strain in detection point 2 under each stress condition.

The concrete structure is cooled by convective heat transfer and heat conduction with air. It takes some time for the concrete structure to drop to the experimentally preset temperature. Thus, the value at which the central wavelength of the FBG reaches stability is taken as the valid measurement value. The experimentally recorded FBG center wavelength offset is substituted into the FBG strain transducer calibration (Equation $\left(8\right)$) to obtain the total strain at the two testing points of the concrete specimen. The thermal strains at the two test points are obtained from the difference between the measured total strains and the applied stress strains. In this paper, the errors of six measurement results at two detection points are analyzed, and the error bar is plotted, as shown in Figure 9 and Figure 10. The maximum relative errors of the test results for the two test positions under each stress condition are 3.87% and 3.56%, respectively. The experimental repeatability is calculated for each test point at each radial pressure condition, as shown in Table 2. The results show that the three measurements are in good agreement. The detection results are linearly fitted according to the least squares method, as shown in Figure 9 and Figure 10. According to the fitting line, it can be found that the slope of the thermal strain fitting line increases as the radial pressure on the structure increases. In this paper, the tensile strain of the structure is taken as positive, and the shrinkage strain as negative.

**Figure 11:**
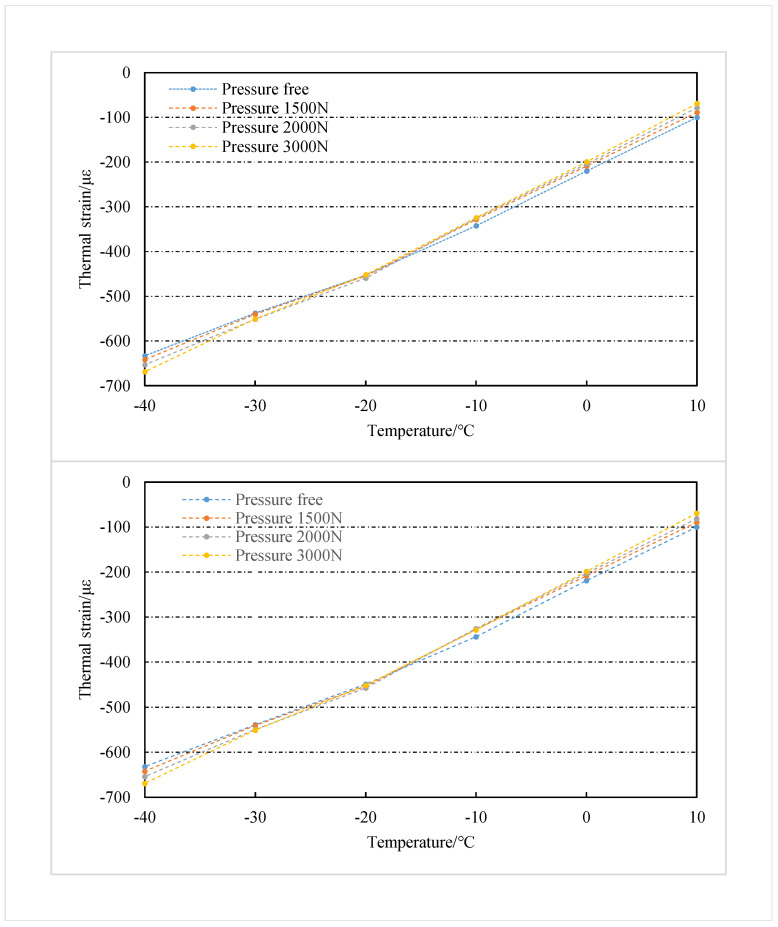
Thermal strain of detection 1 and detection 2 in each pressure condition.

The average value of thermal strain from three repetitive tests is taken for analysis, as shown in Figure 11. The thermal strains of the two detection points increased with the temperature decreased in the temperature range of $-40{\;}^{\circ }C∼20{\;}^{\circ }C$. Compared to the free thermal strain, the thermal strains of the structure subjected to the stress constraint are significantly reduced. It is noteworthy that the difference between the free thermal strain and the thermal strain under each stress condition of the structure decreased with the temperature. Additionally, at the same temperature point, the difference between the free thermal strain of the structure and the thermal strain under each stress condition increases with the stress increase.

The thermal strains of two test points under 3000 N radial pressure are taken for analysis. When the temperature of the two detection points is reduced to $10{\;}^{\circ }C$, the thermal strains at the two detection points under 3000 N radial pressure decrease by 30.9% and 30.65%, respectively, compared to the free thermal strains. The results indicate that stress restraint has a significant inhibitory effect on the cold shrinkage of concrete structures. When the temperature decreases to near $-20{\;}^{\circ }C$, the free thermal strain of concrete and the thermal strain under different stress constraints start to close. This result indicates that the degree of cold shrinkage inhibition of concrete by tensile stress gradually decreases with the enhancement of thermal strain.

### 4.4. Calculation of the Coefficient of Thermal Expansion of the Structure

**Figure 12:**
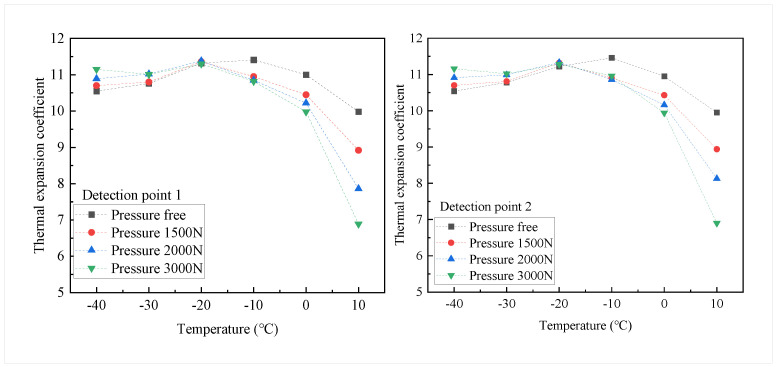
Thermal expansion coefficient of detection point 1 and detection point 2.

Based on the extracted thermal strain of the concrete structure, the thermal expansion coefficient $\alpha_{C}$ of the concrete can be calculated:

(11) $$\alpha_{C}=\frac{\varepsilon_{T}}{\Delta T}=\frac{\varepsilon_{T}}{T-T_{0}}$$

where ΔT is the temperature variation of the concrete structure in the cooling process, *T* is the temperature of the concrete structure in the cooling process, and $T_{0}=20{\;}^{\circ }C$ is the initial temperature of the concrete structure. The thermal expansion coefficients of the two detection points are shown in Figure 12. In the temperature range of $-40{\;}^{\circ }C∼20{\;}^{\circ }C$, the free thermal expansion coefficient of the concrete structure fluctuated around $11\times 10^{-6}{/}^{\circ }C$, with a tendency to increase and then decrease. The deformation fluctuation of the concrete structure during the cooling process may be caused by the freezing expansion of water and the thermal expansion and contraction of ice in the concrete structure [27,28]. The free thermal expansion coefficients of the two tested points reached their maximum at $-10{\;}^{\circ }C$ with $11.41\times 10^{-6}{/}^{\circ }C$ and $11.46\times 10^{-6}{/}^{\circ }C$, respectively. Compared with the free thermal expansion coefficients, the thermal expansion coefficients under the stress constraint condition are significantly reduced. Furthermore, this difference decreases gradually during the cooling process. The difference between the thermal expansion coefficient under the stress constraint and the free thermal expansion coefficient is the largest at $10{\;}^{\circ }C$. When the temperature decreases up to $-20{\;}^{\circ }C$, the thermal expansion coefficients under each stress condition are close to the same.

## 5. Conclusions

An experimental study to analyze the effect of tensile stress constraint on cold shrinkage by measuring the applied stress strain and total strain to extract thermal strain from concrete structures is reported, in which FBG strain sensors were utilized. Two tensile stress concentration zones are created at the intersection of the diameter and the inner ring by applying radial pressure to the annular concrete structure. The FBG strain sensors are fixed along the stress direction in the tensile stress concentration zones. Then, the structure is exposed to low-temperature conditions for gradient cooling, and the applied stress strain and total strain of the structure during the gradient cooling are measured to extract the thermal strain of the structure. The thermal expansion coefficients of concrete structures are calculated for each loading condition. The major findings from this study are summarized as follows:

1. The central wavelength of the FBG for temperature compensation is only modulated by temperature. Thus, the center wavelength of the grating is stabilized rapidly with temperature changes in the experimental chamber. The central wavelength of the FBG strain sensors is modulated by both temperature and structural strain. There is a time delay for the concrete structure temperature to reach the experiment’s preset temperature. Thus, the center wavelength of the FBG strain sensors takes a certain amount of time to reach stability.
2. The thermal strain of the concrete structure gradually increases with decreasing temperature. In the temperature range of $-40{\;}^{\circ }C∼20{\;}^{\circ }C$, the thermal strain of the structure subjected to the stress constraint decreases significantly compared to the free thermal strain. In addition, the thermal strain decrease with increasing stress at the same temperature point. Under the experimental conditions of $-40{\;}^{\circ }C$ and 3000 N radial pressure, the greatest reduction in thermal strain is observed at the two detected points, by 30.9% and 30.65%, respectively.
3. The difference between the thermal strain under stress constraint and the free thermal strain decreases as the temperature decreases. In addition, the higher the tensile stress, the smaller the thermal strain at the same temperature point.
4. In the temperature range of $-40{\;}^{\circ }C∼20{\;}^{\circ }C$, the free thermal expansion coefficient of concrete structures fluctuates around $11\times 10^{-6}{/}^{\circ }C$, with a tendency to increase and then decrease. The thermal expansion coefficient of concrete under tensile stress conditions is significantly reduced compared to the free thermal expansion coefficient. This difference is maximum at $10{\;}^{\circ }C$ and then decreases with decreasing temperature. Under the experimental conditions of $10{\;}^{\circ }C$ and 3000 N radial pressure, the thermal expansion coefficients for the two tested points were $6.99\times 10^{-6}{/}^{\circ }C$ and $6.9\times 10^{-6}{/}^{\circ }C$, respectively.

## Figures and Tables

**Table 1:** Related technical parameters of load sensor.

Measurement Range	Measurement Accuracy	Sampling Frequency	Operating Temperature	Relative Humidity
0–5000 N	0.05%	80 Times/s	$-60\;{}^{\circ }$C$–50\;{}^{\circ }$C	≤85% RH

**Table 2:** Experimental repeatability.

	F=0 N	F=1500 N	F=2000 N	F=3000 N
Detection point 1	1.76%	1.38%	1.46%	1.35%
Detection point 1	1.79%	1.34%	0.98%	1.13%

## Data Availability

Data sharing not applicable No new data were created or analyzed in this study. Data sharing is not applicable to this article.
